# ‘One DB to rule them all’—the RING: a Regulatory INteraction Graph combining TFs, genes/proteins, SNPs, diseases and drugs

**DOI:** 10.1093/database/baz108

**Published:** 2019-11-04

**Authors:** Gianfranco Politano, Stefano Di Carlo, Alfredo Benso

**Affiliations:** Control and Computer Engineering Department, Politecnico di Torino, Italy

## Abstract

In the last decade, genomics data have been largely adopted to sketch, study and better understand the complex mechanisms that underlie biological processes. The amount of publicly available data sources has grown accordingly, and several types of regulatory interactions have been collected and documented in literature. Unfortunately, often these efforts do not follow any data naming/interoperability/formatting standards, resulting in high-quality but often uninteroperable heterogeneous data repositories. To efficiently take advantage of the large amount of available data and integrate these heterogeneous sources of information, we built the RING (Regulatory Interaction Graph), an integrative standardized multilevel database of biological interactions able to provide a comprehensive and unmatched high-level perspective on several phenomena that take place in the regulatory cascade and that researchers can use to easily build regulatory networks around entities of interest.

## Introduction

Genes regulation in eukaryotic cells is driven by a large number of complex interactions that take place among several regulatory entities, which may belong to different categories such as sequence-specific transcriptional/posttranscriptional regulators, DNA-binding proteins, coactivators and chemical interactions. Such a heterogeneous and wide variety of regulators act in concert to control or tune the expression of each single gene. In the last decades, literature mining and experimental studies have helped researchers to discover and understand several of these regulatory interactions and to infer from them a significant number of regulatory subnetworks (pathways), made available through several public databases (1) (2).

However, while each database is a very specialized source of data, researchers often encounter difficulties when integrating data from different repositories. There are several reasons to this, and here we discuss the ones that in our opinion are the most relevant.

### Scope

A large number of the available databases limit their scope to only one or few types of interactions. Many databases take into account transcriptional regulation only, while neglecting posttranscriptional mechanisms; others provide links to only a limited subset of homogenous interactions, thus lacking a holistic perspective on the complex set of heterogeneous interactions that very likely co-occur in a more realistic regulatory model. For instance, protein–protein interaction (PPI) databases usually map and possibly assign a score to each physical interaction and functional association. However, interactions reported in these databases (like Mentha (3), String (4) or Fisingene (5)) only refer to interacting proteins; thus, the resulting interactome remains somehow incomplete, unless other important molecular interactions are included. As another example, microRNAs (miRNAs) databases, such as miRTarBase (6) and Targetscan (7), contain only specific miRNA–mRNA interactions, and they are not usually included in the context of pathways and signaling cascades. We already addressed this problem in Politano *et al.* (8) (9). Similar limitations apply to databases related to intergenic or intragenic miRNA ontogenesis, like miRiad (10), whose aim is to provide a direct link from host genes to their cotranscribed miRNAs, thus lacking any other information regarding possible interactions among the hosting genes.

### Data format

The fact that search results are often provided as a batch download of large custom structured plaintext lists with proprietary formalisms and naming conventions results in an overall small data interoperability and high management complexity (11). As an example, PPIs usually include hundreds to thousands of regulations. Such a large number of interactions, often reported in terms of a plaintext list, is quite difficult to handle in its raw format. Building parsers for these data is often a time-consuming and error-prone task, and information retrieving from these sources of data may result inefficient. Things get even more complex when we consider other sources of regulation, like transcription factors (TFs) (12, 13) (14) and coTFs (15), as well as drugs (16, 17, 18), other chemicals (19, 20) or genetic variations [like single-nucleotide polymorphisms (SNPs) (17)] possibly interfering with, or modifying, the normal regulatory behavior.

### Data sources

Synchronism in data integration is another problem. Due to asynchronous updates in data sources, direct linking in databases may rapidly become obsolete and, even more dangerously, source of erroneous assumptions. For instance, the StarbaseDB, which is indeed a valuable source of data, was only recently updated. Before this last update, however, the maintained catalog of miRNA IDs was referred to miRBase Release 20. This was a significant source of problems, since miRNA IDs have been largely renamed and reassigned in miRBase Release 21, which has been remapped against the new human genome assembly, GRCh38. During this update, miRBase curators cleaned up dubious and misannotated sequences and reassigned previously used ids. The result, according to the differential changes between Releases 20 and 21, is that 169 hairpins and 353 mature sequences have changed names (21). Therefore, any work citing sequences belonging to Release 20 may currently refer to different and unexpected miRNAs. Keeping track of the consistency of cross-references among different databases is not trivial and must be taken into account every time data from multiple sources must be integrated.

### Naming and standards

On top of the previously discussed limitations in data integration, to make things even more complex, the overall lack of unified standards and naming convention makes it often really hard to properly cross-match information among multiple data sources (e.g., see the lookup table available from Unichem (19), which provides for each chemical up to 35 different aliases that show how there is no consensus in uniquely identify each chemical). Despite that integrative databases (DBs) have been built in the past years, the actual results are still limited to very specific domains (22) and usually provide only a limited set of interactions (23), thus resulting weak from a holistic perspective.

The obvious solution to these problems would be to have a set of widely accepted international standards to regulate data formats and naming conventions. Unfortunately, so far there is no consensus in the field of life sciences researchers, thus leading to several overlapping and sometimes conflicting conventions adopted in different communities.

Therefore, since standardization is still far from being a viable solution, and given the overall need to provide life scientists with a holistic representation of all the complex interactions that simultaneously take place in complex genetic regulations, in this article we present the RING (Regulatory Interaction Network Graph), a unified, holistic and standardized data repository integrating data from 38 different sources. The RING has been constructed in such a way to resolve naming inconsistencies and to present the resulting data at different levels of detail and aggregation.

The RING is the result of a more than 1-year-long effort that is now able to offer a holistic representation of regulatory interactions based on heterogeneous publicly available databases.

In particular, the most attractive features of the RING database are as follows:

- **Scope:** It integrates regulatory interactions information about TFs, coTFs, miRNAs, chemical compounds/drugs, diseases, polymorphisms and target genes/proteins, as well as a very large amount of predicted or experimentally observed transcriptional and posttranscriptional interactions, from 38 selected databases. Its overall structure is designed to guarantee further extensions to other regulatory entities.
- **Naming and standards:** It provides nomenclature standardization (of genes, proteins, miRNA names and all types of interactions between them), in order to make multiple sources of data reliably cooperating together; for example all same/similar interaction types, named in different ways in the original databases, have been translated according to a common dictionary.
- **Data sources:** To guarantee the highest data-integration reliability, we carefully analyzed the structure and organization of all data sources and, for each of them, created automated pipelines able to continuously maintain data synchronization and up-to-date cross-reference nomenclature. The inclusion of new sources is possible and will be a continuous process. Obviously, each new data source will not be immediately integrated ‘as-is.’ Each new data source will have to be verified for compatibility with the data already present and then imported using custom automatic procedures.
- **The RING graph:** It exposes its data in the form of a preassembled repository of complex regulatory interactions. In the current version, the RING includes a regulatory network model composed of almost 75 k interactors and 1G interactions.

The RING is not the first attempt of integrating large amount of biological information. We are aware that there is a plethora of large integrated databases [e.g. NCBI (24), University of California Santa Cruz (UCSC) (25), etc.], each of them containing both peculiar and unique but also overlapping information. With the RING database, we do not want to claim that these databases are not useful. We are instead focused on building an integrated database that tries to merge the common knowledge of all these data sources into a single repository specifically oriented to the interactions of interest in regulatory networks. Peculiar information of each database used to build the RING database remains extremely important, and for this reason, the RING database maintains a link to each original data source, thus guaranteeing the completeness of the information.

The RING was not developed with a precise biological question in mind. The general idea was to provide a tool that allows an easier exploration of the heterogeneous regulatory interaction networks in the human genome. Basically, it is a tool that life sciences researchers can use to build regulatory networks around entities of interest. It was not our purpose to create a tool to solve problems that cannot be solved in other ways. The RING integrates data from publicly available sources, so every result obtained with the RING could be obtained, with a much more time-consuming and laborious process, querying each of the individual databases and selecting, at each step, the data of interest. Nevertheless, this is the same also for other ‘large integrated databases,’ which do not allow to solve otherwise unsolvable problems, but simply make knowledge extraction easier and faster. In the additional material, we added two use-cases that demonstrate two possible scenarios in which the RING database could be used.

**Figure 1:**
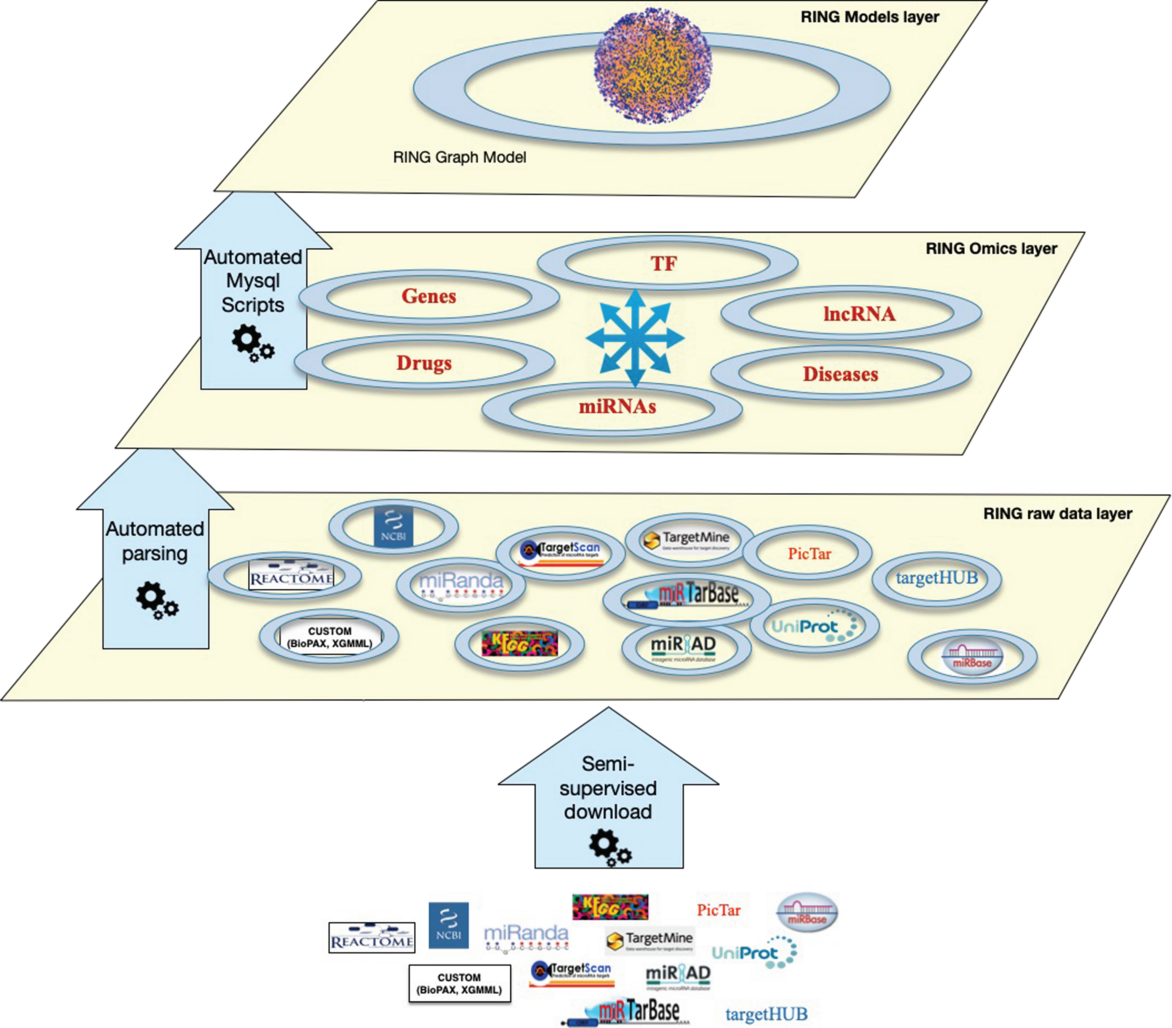
The RING architecture. The database is organized into three hierarchical layers. The bottom layer (raw data layer) integrates raw data imported from several external sources, the middle layer (omics layer) groups together data referring to similar interactors, and finally the top layer (model layer) exposes data in a holistic network-like representation.

## The RING architecture

The RING database is organized in several layers, each with an increasing degree of data integration (Figure 1):

- **Raw data layer:** This is the lower layer, where each individual data source is automatically downloaded from its online repository. Each data source goes through a standardization process of its internal naming references. This allows us to uniform the data representation by resorting to a limited set of allowed naming authorities. Details and implementation are reported in the Overall Naming Conventions subsection.
- **Omics layer:** This is the middle layer, where data referring to the same interactors are grouped and integrated together. This layer also integrates all interactions data between pairs of interactors.
- **Model layer:** This top layer exposes data in a holistic way. In the current implementation, the RING model layer includes a RING graph model representing all interactors and interactions available at the omics layer. The RING graph is organized, for performance issues, into three tables, the first dealing with genes–miRNAs–TFs interactions, the second with diseases–genes–SNPs associations and the third one with drugs–genes–SNPs associations. To create this network, to our knowledge unique, the RING authors defined a vocabulary to standardize both naming conventions as well as all possible interaction types.

Each data source will be synced and updated whenever a new release is made available and is compatible with all the ‘integration’ requirements in terms of consistency and availability of naming convention lookups. The inclusion of new sources is possible and will be a continuous process. Once the import procedure of a new data source is developed, data can be automatically kept in sync as done with the data sources already considered.

In particular, an automated pipeline based on Python can be configured for different synch tasks with different schedules. The pipeline is currently able to automatically check for updates of most of the data sources (the ones that maintain a programmatical way to download updates); a specific configuration may be required in order to update other sources or to add new data shapes.

The work done here was to create common dictionaries and translation rules in order to have a uniform representation of all data. The outcome of this activity was the creation of an automatic procedure that allows us to maintain the RING database in sync with the original sources through periodic updates.

Apart from the automated backend, which procedurally processes raw data to be included or updated in the database, we designed an interactive web-based user-friendly application layer allowing to query and extract knowledge from the integrated data, download reports and analyze results. This latter layer is in continuous expansion as we implement advanced network analysis functionalities.

### Raw data sources and the omics layers

The RING is a relational database built with particular attention to the concept of ‘relation’ among omics entities (the interactors). Both the omics layer and the model layer have been built starting from the data available in several publicly available databases. The following subsections detail each source.

• **Genes and predefined gene regulatory networks:** Gene information has been downloaded from NCBI (24) and UCSC Genome Browse (25). Basic information has been retrieved from NCBI for genomic assessment (i.e. naming, geneID and aliases extraction for further use in naming standardization). The UCSC Genome Browse has been accessed via its MySQL interface to extract gene locations expressed as start/end base pairs and chromosome, and then it has been further used for the identification of putative intergenic miRNA hosts. Gene regulatory data usually represented as pathways have been obtained from KEGG (2);
• **TFs and co-TF interactions:** Transcription factors and coTF regulations have been extracted from TcoF-DB (15), TargetMine (14), tRRust (12) and tf2dna DB (26) (we included all the nine experimental subsets (13, 26–33), as well as the full set of computationally inferred regulations. Transcription factors data are usually provided in the form of TF- > regulated gene relations. Besides this commonality, different databases may provide, as extra attributes, the binding scores (i.e. scores that represent binding free energy or some similar binding strength score) and further interaction details like the transcriptional effect TF has on its target (i.e. repression or enhancing). All scores are maintained as raw data, but their integration into a unified score is still under evaluation.
• **Protein and protein interactions:** Protein information and naming standardization have been collected from UniProt (34). Other data sources have been used for accession lookup; in particular, we exploited the cross-references data between Uniprot and ChEMBL (20), Stitch (35) and String (4). Since multiple Uniprot ID (UID) are currently associated to the same gene [for instance, A1CF gene has currently associated (among the others) some isoforms like UID: A0A024QZJ5 and UID: A0A024QZM7 and also similar coded/pseudo protein like UID: B4E1E3], for sake of simplicity we annotated all the isoform proteins encoded by the same gene, with the name of their host gene (in this way, each gene represents the cluster of its coded proteins). Protein–protein interaction data have been extracted from Fisingene (5), Irefindex (36), String (4), Mentha (3), Reactome (1) and Signor (37). There is a significant overlap of information among these sources. Single PPI interactions/regulations are in fact usually expressed as a linkage between two proteins, along with extra attributes that further explain the interaction. Some data sources may offer a more or less formal vocabulary of heterogeneous regulatory terms. Where available, all regulatory terms have been inspected to extract further regulatory information (in particular the interaction effect, i.e. repression or enhancement) and used to build a custom controlled vocabulary of interaction types (see The RING graph). Whenever a straightforward and clear interpretation of the interaction effect was not possible (like in the case of a generic ‘physical association’ attribute), we just reported the association as ‘undirected.’ Whether or not the interaction effects can be extracted and normalized in our dictionary of terms, the original label is always available to the user for a more meaningful visual assessment of the results. Among the additional data sources, we especially appreciated Signor (37), a collection of approximately 12 000 manually annotated causal relationships between over 2800 human proteins participating in signal transduction. The causal relationship guarantees that data coming from such repository always have a clear interaction effect and a clear direction for each entry, as well as some bibliographical references that experimentally confirm the regulation.
• **miRNA ontogenesis and targeting:** MiRNAs information has been collected from MirBase (21) for basic posttranscriptional assessment and naming standardization. In particular, we resorted to mature miRNA accession ids as general database naming convention. When only single precursor miRNA references were available (as in miRNA ontogenesis DBs), we specifically accounted the regulation to the two mature forms of the miRNA, if both exist in MirBase.
For miRNA ontogenesis, we resorted to miRIAD (10), a DB that contains cotranscriptional effects that take place between miRNAs and their intragenic and intergenic host genes. MiRNAs may be in fact located in intergenic regions (‘intergenic miRNAs’) or mapped to intragenic loci of protein coding genes (namely ‘host genes’). Another custom approach to further and broadly infer other miRNA host genes has been implemented by looking at miRNA coordinates reported on MirBase and UCSC; in particular, we searched for all possible miRNAs whose genomic coordinates fall into the genomic coordinates of a surrounding gene (i.e. intronic or intragenic miRNAs), and miRNAs that are not located into any intronic region are instead reported along with their closer upstream or downstream gene. This may be meaningful according to the fact that the expression of intergenic miRNAs has been reported affected by their genomic context. In França *et al.* (38), the authors, focusing on miRNA neighbor coding genes, discovered that intergenic miRNAs are distant from a few dozens to >1.5 Mb (median = 34 kb) bases. Furthermore, according to our knowledge, this kind of data has not been previously reported in any publicly available data source, which makes the RING the only DB actually reporting closer neighbor genes (inferred hosts) on a miRNA-wide basis.
For miRNA targeting, we resorted to data available on mirTarBase (6), MirWalk (39) and TargetScan (7). Mirtarbase is currently the largest source of validated miRNA–target interactions, which makes it a reliable source of miRNA regulatory information, and also largely reduces the usual and often unmanageable number of inferred targets. Such approach may be useful to rapidly confirm preliminary hypotheses or select best candidates for experimental procedures according to robust previous knowledge. For more exhaustive and possibly new discoveries, the list of targets may be enlarged by resorting to computationally predicted miRNA–target interactions available in both MirWalk and TargetScan. The RING integrates all these sources by providing a unified view of all miRNAs interactions with their regulating/regulated entities.
• **SNPs:** dbSNP (40) has been used to extract SNP basic information as well as to define an SNP naming standardization. For each SNP, we collected the dbSNP id, the prognostic role and the name of the gene affected by the polymorphism itself. SNP interactions have been extracted from DrugBank (17) that reports drug–SNP interactions and from pharmGKB (18), which includes both SNP–disease and drug–SNP linkages.
• **Drugs and chemicals:** Drugs basic information has been collected from ChEMBL (20) and UniChem (19). Whenever possible, drugs have been referred and conventionally named across the DB with their ChEMBL id and their accepted name. Nonetheless, given the amount of chemicals not included in ChEMBL, we also resorted to UniChem as a second naming authority. Given its open approach, UniChem allows the community to include their own chemicals in the DB, resulting in a larger collection that contains any possible chemical. On the other hand, UniChem is much more prone to reference errors since it allows for duplicated information and ambiguous references (e.g., there are several ids that point to the same chemical form), which may fragment further integration and/or require a large supervised effort to be fully understood. Drug interactions have been extracted from DGId (16), DrugBank (17), PharmGKB (18) and STITCH (35). The drug interactions collected so far include drug–gene, gene–drug and drug–drug associations.
• **Diseases:** Diseases basic information has been collected from OpenTargets (41) and DisGeNET (42). As for drugs, given the lack of a single naming reference, we resorted to a two-level naming convention. Given the largest amount of diseases is reported in DisGeNET when compared against OpenTargets (i.e., 10 053 diseases in OpenTargets and 13 074 in DisGeNET), we chose DisGeNET as the primary reference. Whenever possible, diseases annotated in other sources have been remapped against their DisGeNET id and, if failing, against OpenTargets. Diseases not included in one of the two databases have been flagged, maintained in the database and associated with the original name provided in the evidence. Disease interactions have been extracted from DisGeNET, OpenTargets and PharmGKB (18) and represent association between disease and genes/SNPs.

### The omics layer

The omics layer aims at standardizing and reorganizing the data collected in the raw data layer in order to structure the available information and to make it easily accessible. However, the RING DB does not want to replace the original databases. While the RING DB puts its main focus on organizing information about interactions among the considered entities, each data source includes a large amount of peculiar information that could have significant value depending on the specific biological question. For this reason, we decided to keep cross-reference links to the original data sources. In particular, each database entry is provided with a direct link to its original data source using a unique reference to a cross-reference table storing all the extra information about each data source. This is helpful to link back results to their original sources in order to allow the user to get more details on a given interaction or interactor. Each entry is also linked to the specific row of the raw tables used to compile the RING. This hidden treasure of information easily allows us to augment the amount of data integrated in the RING and possibly build better knowledge extraction models in its future releases.

The SQL dump of the omics layer tables is available in the ‘Download’ section of the RING website. In this way, researchers will still be able to take advantage of the data without being dependent on the web interface performances.

As mentioned in the previous section, each interactor present in the RING database has been mapped against one or more reference databases in order to provide common accessions. A lot of curation effort has been in fact spent to build the lookup tables necessary for a reliable cross-database translation of all interactor names and ids. As a result, all naming conventions are consistent through the whole DB, no matter the original source of data. Table 1 summarizes, for each interactor type, the source of data we adopted for its names catalog. In this way, search results may be easily enriched by directly linking each interactor to its original reference DB and possibly integrating extra information that goes beyond the scope of this release of the RING, but may be useful for more effective comparative capabilities and human-supervised assessments.

**Table 1:** Naming authorities

**Entity**	**Naming source**
Gene	id (NCBI), symbol (HGCN)
TF	id (NCBI), symbol (HGCN)
Protein	id (UniprotKB)
MiRNA	mature mirna id (miRBase)
Drugs/chemicals	id (cheMBl), id (UniCHem)
SNP	id (dbSNP), symbol (HGCN)
Diseases	name, id (DisGeNET), id (OpenTargets)

**Figure 2:**
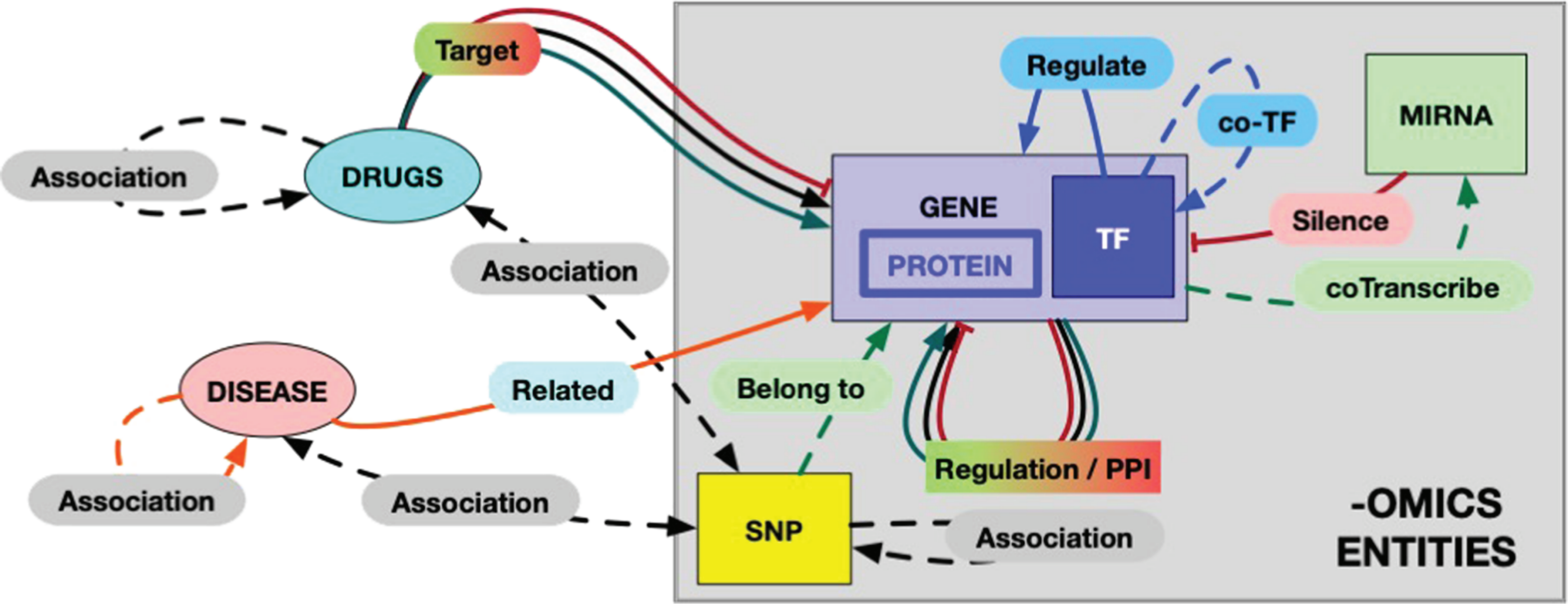
The RING model schema. Each entity and each possible interaction is shape- and color-coded in the graphical result provided by the RING web interface. The color codes are defined as follows: red = inhibition, green = activation, black = undirected interaction, blue = TF coregulation, orange = disease–gene relation. Dotted lines represent weaker association, while solid lines represent more reliable (possibly causal) interactions. In particular, double-headed arrows represent undirected relations to account for interactions that rely on the concept of ‘association’ instead of ‘causality/targeting’ (like snp/gene–drug relations). To the best of our knowledge, this would be a safer approach to avoid interpretation error.

## The RING graph

One of the primary motivations to build the RING was the desire to create a homogenous network-like representation of the interactome and to make it compact, reliable and fast enough to be a useful instrument both for computational and human-supervised approaches.

The overall schema of this model, called the RING graph, is summarized in Figure 2. Each entity and each possible interaction are shape- and color-coded in the graphical result provided by the RING web interface. Hopefully, this formalism renders a more readable picture by allowing to easily identify same interactor types. All omics (genes, proteins, coTFs and SNPs) interactors are represented with rectangular symbols, while their colors differ according to the specific family they belong to. miRNAs, drugs and diseases are instead represented by oval symbols. It is also possible to filter in and out specific edges in order to switch among dense and light views, thus guaranteeing the ability to tune the preferred amount of details while avoiding unnecessary and possibly confusing data.

**Figure 3:**
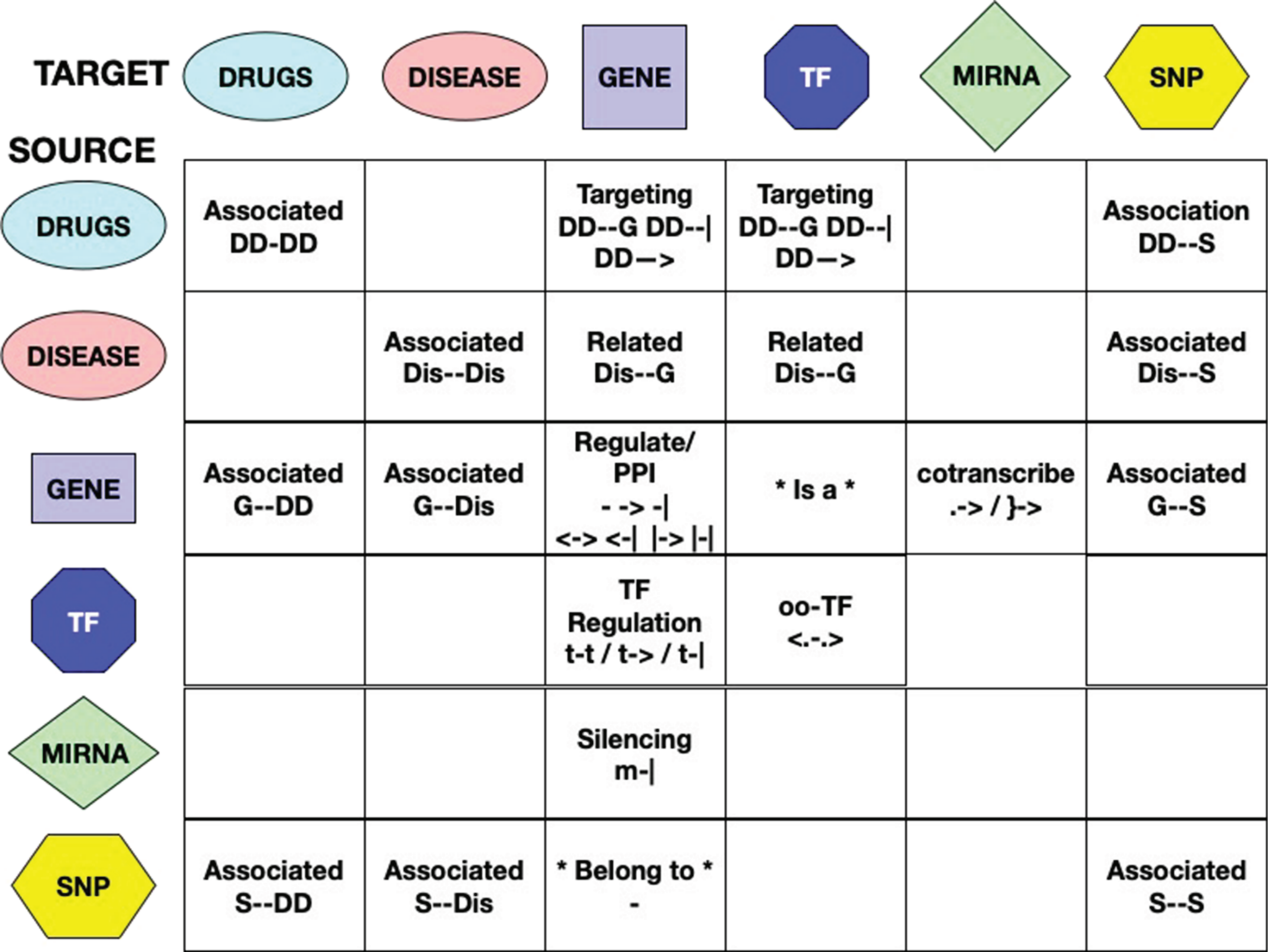
Interactions among entities—meaning and unified symbols. In order to provide a normalized dictionary of high-level interaction types, the RING provides a set of symbols hereby defined as *direction.* The figure reports all the dictionary symbols organized as a table in which rows represent source entities and columns represent target entities. Empty cells represent interactions that are not currently available in the RING because no reliable data sources of that type have been identified. For available interactions, instead, the table reports its normalized set of *directions* and their overall meaning.

Interaction types have been uniformed, whenever possible, in a manner similar to the one used for the interactors naming standardization. For each interaction, the RING database uses three custom fields: *direction*, *action* and *score* to uniformly represent all possible types of relations. The *direction* field reports a unified dictionary of symbols able to largely uniform the network representation in terms of the interaction biological meaning (Figure 3). This field has been manually verified for each included source of information in order to translate their custom/proprietary naming conventions into a general representation. The *action* field reports the original annotation that was used to define the corresponding symbol. This field is especially useful to further disambiguate undirected relations. For example, the same direction symbol for undirect relation (‘-’) may be applied to both ‘physical association’ and ‘complex input’ actions. We chose to text-code symbols instead of assigning them a numerical id in order to make this information more easily human-readable directly from the database search results.

The score field reports, where available, the confidence values inherited from each database. The main problem in their integration is that their values, depending on the original source, may have different scales, meaning and ranges. We are currently evaluating possible ways of normalizing these scores in order to make them work together.

## Accessing the RING

The RING database can be accessed through a user-friendly web interface available at https://precious.polito.it/theringdb.

The biggest challenge in creating the web interface to access the data of such a large database has been to trade off between performances and data granularity. Queries returning too much data are useless because they may require too much time to complete or result in networks that are too large to be of any realistic use. For this reason, the web interface provides a wide set of filters that allow the user to precisely select the type of interactions that should be targeted in each query. Queries that would possibly return too many interactions are not allowed. All the available data are nevertheless available, giving the user the ability to individually analyze all information of each single interaction present in the network (see the Omics layer section). This does not mean we are not available to provide more extensive bulk ‘custom’ interaction data to researchers who may request it.

Nevertheless, we believe that the available web interface will allow, with a short learning curve, life science researchers to have an unprecedented user-friendly access to a huge data set of regulatory interactions.

### Filtering data sources and interaction types

**Figure 4:**
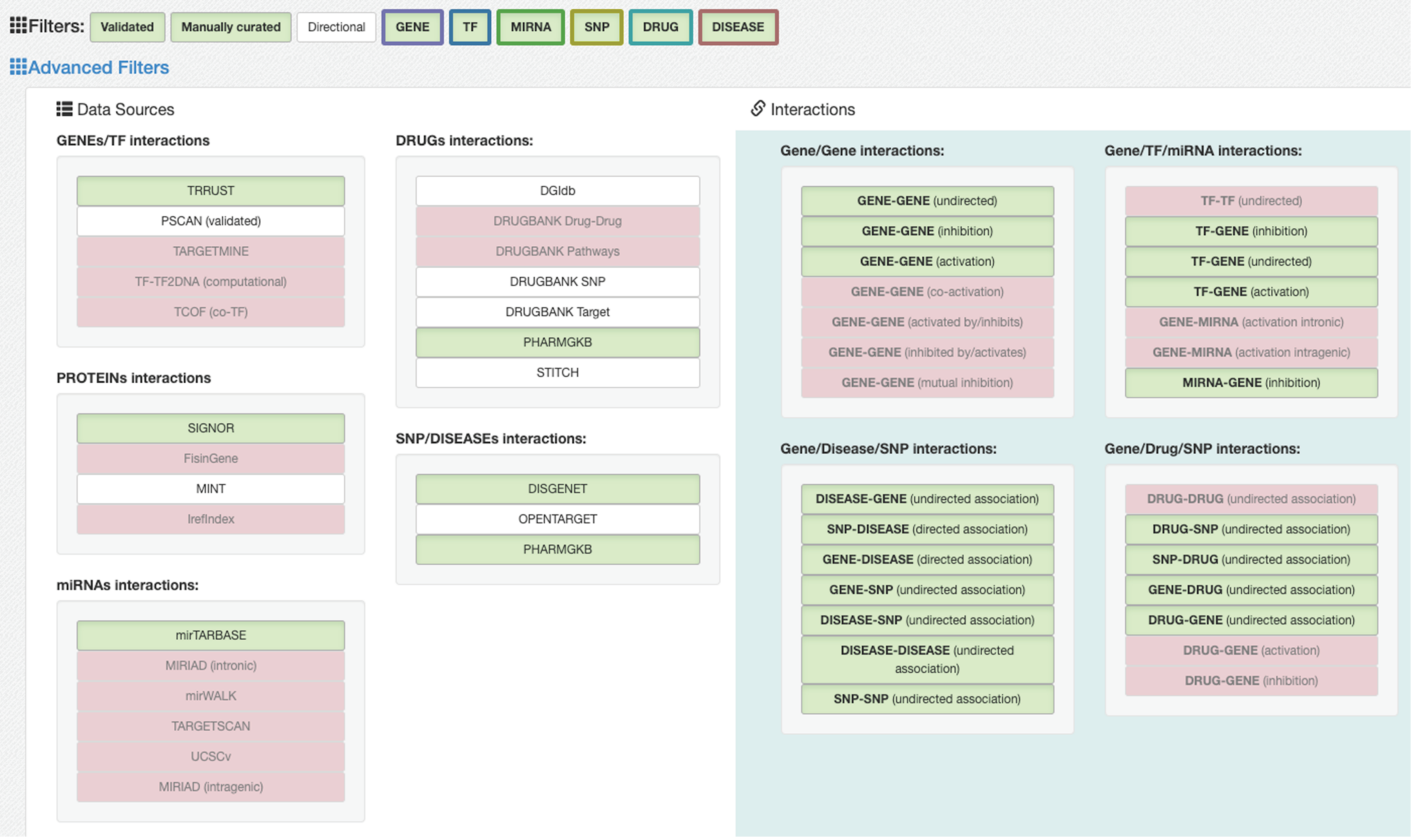
Data sources filtering panel. Available basic filters are divided into two groups: (i) Validated, Manually curated and Directional that filter the list of data sources to query according to their overall reliability level and (ii) the set of entity-related filters (i.e. GENE, TF, MIRNA, …) to choose what types of entity should be included in results. With the first group, the user can select the desired confidence level, trading-off between minimal but reliable data (i.e. only curated interaction for reducing the cost of an experimental setup) or larger but possibly unreliable results (i.e. to identify possible unexpected regulator not yet confirmed experimentally). The second group allows the user to include all the entity types available or possibly remove unwanted entities in order to reduce the result complexity (i.e. by excluding from queries all the drugs). Furthermore, a set of progressively more detailed filters is available in the Advanced Filters section. This helps in further fine tuning the DB interrogation and helps users to select (i) specific databases listed in the Data Sources section, to limit results to only a subset of the available sources, and (ii) specific regulatory directions available in the Interactions list, to filter results according to the type of interaction [e.g. a user may be interested only in antagonist TFs targeting a given entity; thus, selecting only ‘TF-GENE (inhibition)’ should be the best option to only retrieve those information largely reducing the amount of unwanted information].

Before querying the database, it is necessary to select the desired data sources. The source filtering panel (Figure 4) is made of a set of basic filters and an ‘Advanced Filters’ section. The basic filters, include a set of buttons (‘Validated,’ ‘Manually Curated’ or ‘Directional,’ ‘Gene,’ ‘TF,’ ‘miRNA,’ ‘SNP,’ ‘Drug,’ ‘Disease’) that allow the user to activate/deactivate different sets of predefined data sources.

The advanced panel is divided in two sections, each with an increasing level of detail. The first column allows the user to further refine the selection/deselection of the individual data sources. When a network is loaded into the system, the number of interactions originating from each individual database is reported in parenthesis next to each database name. The second column allows the user an even more detailed filtering of the types of interactions present in the selected databases. To avoid the selection of incompatible filters, the filtering buttons are linked together so that a click on a filter button possibly enables/disables other buttons in the other columns.

### Data input, network validation and basic network operations

**Figure 5:**
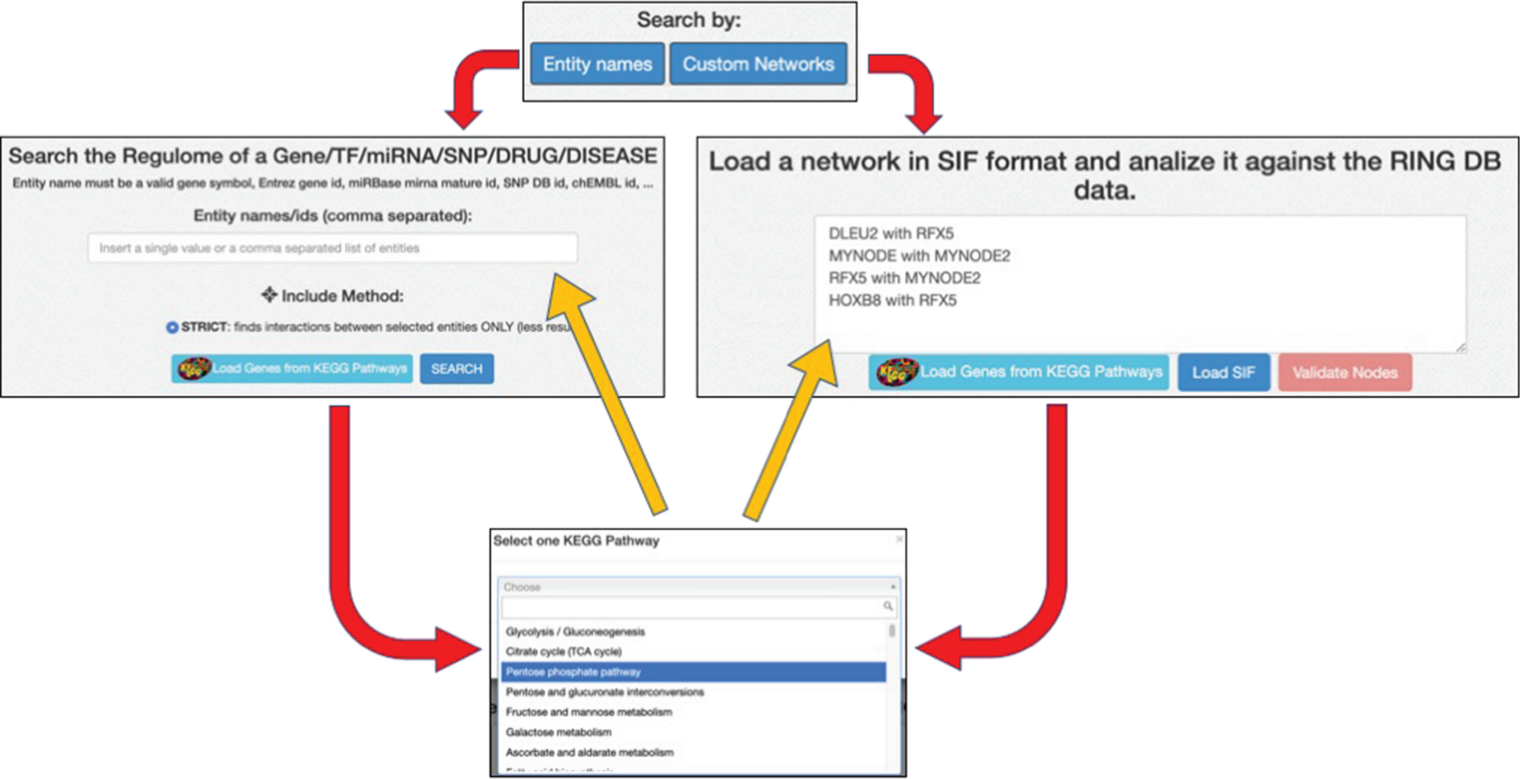
Data input option. The ring has two main search procedures: (i) by comma separated entity names, (ii) by loading a SIF network. Both the procedures may use precompiled set of interactors taken from KEGG pathways. Regardless of the selected input method, the names of the input interactors are validated against the interactors present in the RING database. Each interactor is colored and shaped according to its type (gene, TF, miRNA, SNP, drug, disease or unknown).

Each of the two available ‘Search By’ buttons opens one of the panels shown in Figure 5. In the ‘Entity Names’ panel, it is possible to start with a simple comma-separated list of interactors. The ‘Custom Networks’ panel instead allows to load a network described in SIF format (http://www.cbmc.it/fastcent/doc/SifFormat.htm). In both cases, it is possible to populate the input fields with the names of the interactors of a KEGG pathway.

When the network is generated starting from the entities names only, the RING database is queried for all the available interactions (filtered according to the selected data sources) between the selected nodes. Interactions of the input entities with other entities present in the database (but not in the list of queried nodes) are excluded because they would otherwise result in an unmanageable number of results.

**Figure 6:**
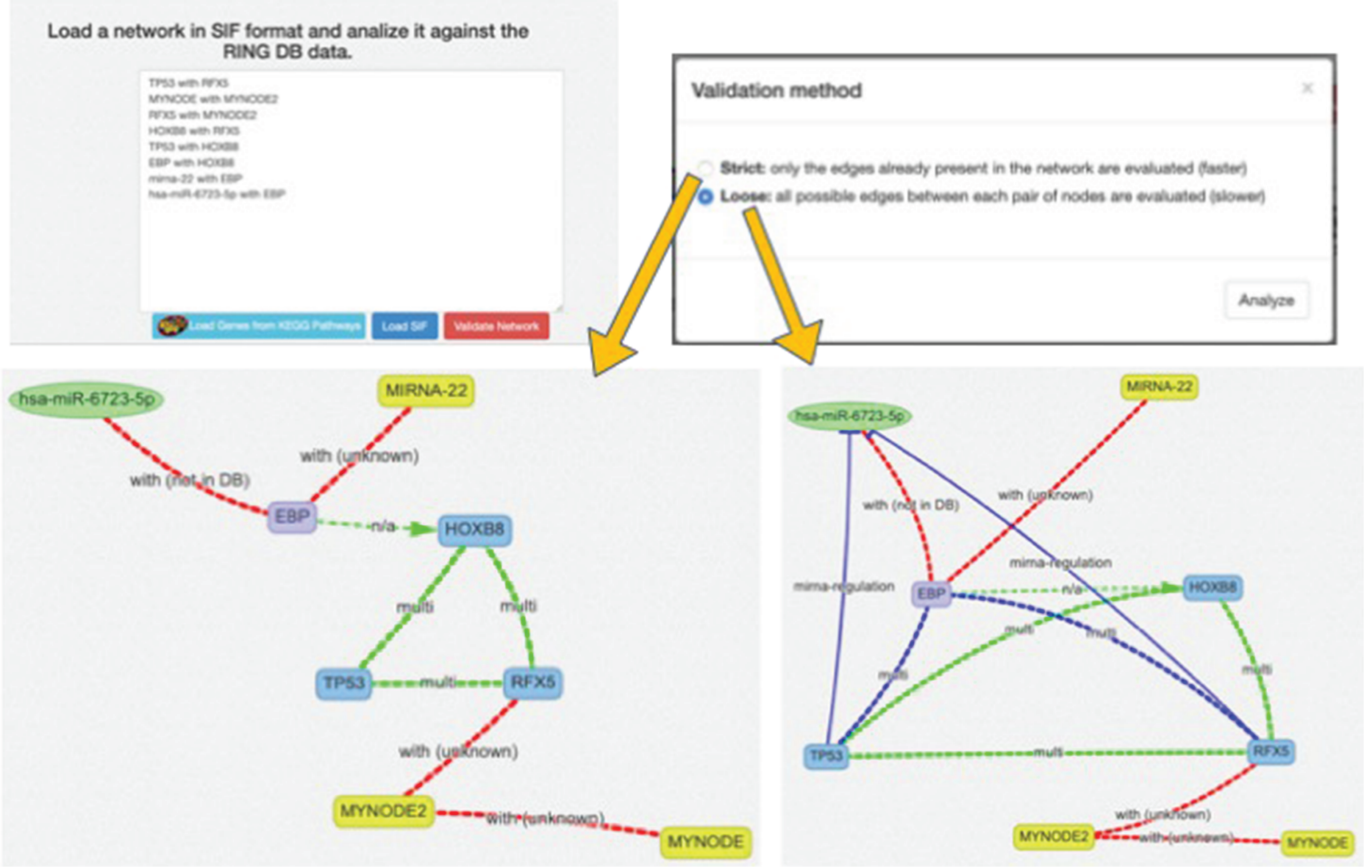
SIF and interactors validation. This panel offers a text area to insert a SIF network description, which is particularly useful to rapidly design or import custom networks in the web interface. The SIF description is in the form of <SOURCE_ENTITY> < ACTION_TYPE> < TARGET_ENTITY>. When the SIF network is loaded, all the valid entities, provided with accepted names, aliases or accessions, are automatically recognized, and their naming is normalized according to the RING naming conventions. Unrecognized entities are highlighted in yellow color. The loaded network loaded may be further validated in terms of interactions, thanks to the Validate Network procedure, which exploits the RING knowledge in order to also confirm if the interactions described in SIF belong to the current knowledge and possibly if some connections are missing.

When the network is generated starting from a custom or predefined SIF file, the network validation button allows the user to validate each node and each edge of the network against all the available data sources (Figure 6). This can be done using the ‘Strict’ option, where only the edges in the original network are validated or ‘Loose,’ where all possible connections between the network nodes are evaluated (and added, if missing) in order to possibly discover new interactions. New interactions are colored in blue, whereas interactions not present in any database are colored in red and annotated as ‘not in DB.’ By clicking on an edge, it is possible to retrieve all its existing information. In this way, researchers can easily verify if the interactions in their network are supported by data. To the best of our knowledge, this functionality is not available in any other publicly available resource.

**Figure 7:**
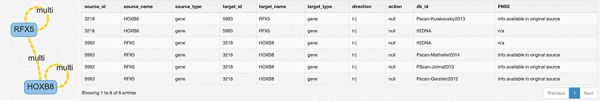
Multiple interactions. When multiple interactions between two entities are present in the same or another database, they are represented in the RING as a *multi* edge colored in yellow. To better understand the overall meaning of such edges, it is possible to click on them to retrieve a detailed table, displayed below the network, which reports all the details on the interaction of interest. This annotation becomes particularly important when different databases report different interaction directions; e.g. a TF may be reported as enhancer and silencer of the same target in different DBs.

**Figure 8:**
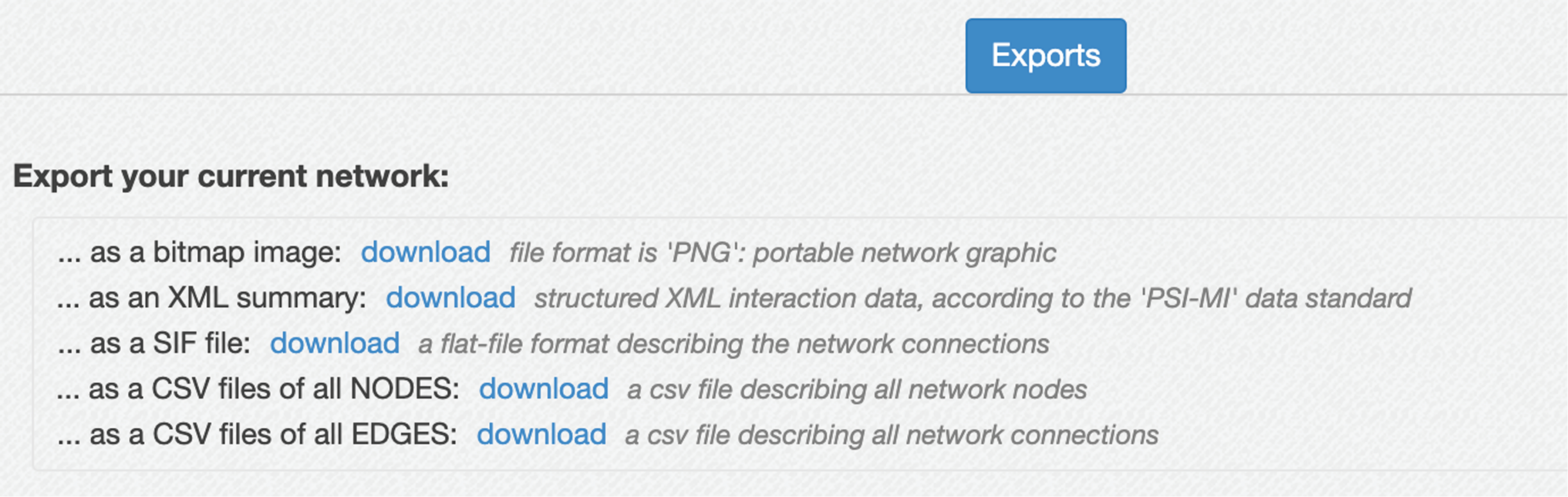
Network export interface. The interface provides several ways to download the network created in the RING. The export menu allows users to save current results as a PNG image, in network formats like SIF and XML that provide easy data exchange with other network analysis tools (custom python script, Cytoscape, Gephy, etc.), and both the node list and the edge list as csv files. Those latter files also contain all the extra attributes, usually not included in the network representation but currently available in the RING, to maximize the informative content returned.

**Figure 9:**
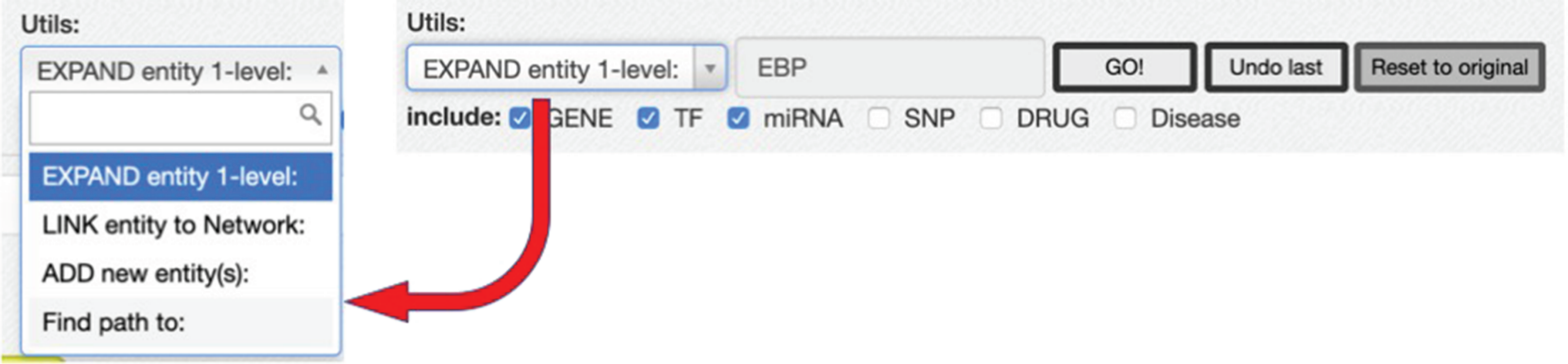
RING graph interface: the *Utils* panel. The text area allows users to insert a regulatory entity or a list of entities separated by comma, which may be new or already included in the network. On top of them, different procedures may be applied according to the select box choice. The ‘ADD new Entity(s)’ procedure simply includes the new entity(es) in the network as nodes, if they are not present already; the ‘LINK entity to Network’ searches for the specified entity’s interactors already present in the network and links it to them, and finally, the ‘EXPAND entity 1-level’ searches for all the interactors of the new entity and provides a dialogue window (Fig. 10) to further refine the expansion procedure.

After the network is loaded and visualized, three sets of buttons allow the user to choose the network layout, to hide/show nodes’ groups and to cluster nodes according to different criteria.

When multiple interactions are present between two interactors, the connecting edge is labeled as ‘multi’ (Figure 7). By clicking on it, a table is displayed below the network detailing all interactions available in the complete collection of databases (in this case, the data sources filter is not applied).

Below the network area, an Export panel (Figure 8) allows the user to export the current network information in different formats for further elaboration.

**Figure 10:**
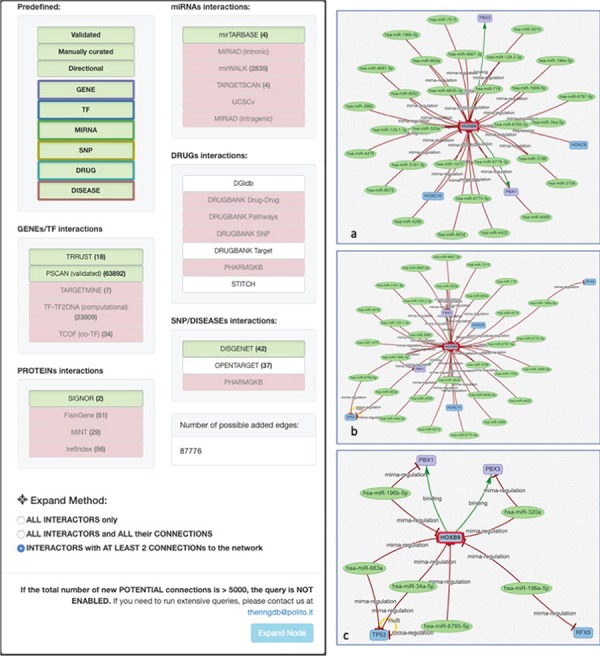
Expand mode panel. It provides predefined buttons that provide filtering capabilities at entity level and curation level. More detailed filters, available for each data source, allow to discriminate at source database level. They also provide an estimate of possible interactions belonging to the selected entity and possibly added to the network. The Expand Method radio buttons make it possible to select one of three expansion methods. ‘ALL INTERACTORS only’ method will only add the interactions between the selected node and its interactors (Figure 10a); the second will allow to also add the interactions between the node interactors and any other node present in the network (Figure 10b). The last option will run an additional routine that will keep only those interactors that have at least two interactions with any other node of the network (Figure 10c).

#### Network expansion

After loading the initial network, it is possible to start expanding it using the *Utils* panel (Figure 9). Three main functionalities are provided: ‘ADD new Entity(s),’ ‘LINK entity to Network’ and ‘EXPAND entity 1-level.’

To add a new interactor, it is enough to write the name (or comma-separated names) of the interactors to be added, and after they are validated against the database available interactors, they are added to the network as isolated nodes. The LINK and EXPAND functions are applied to any node of the network that is selected (by clicking on it). The LINK functionality searches for all possible interactions between the selected node and all the other nodes already in the network. The EXPAND option is more complex because it attempts to find interactions with other entities not already present in the network, and this search could potentially return a very high number of results. For this reason, after launching the EXPAND procedure, a new panel is displayed (Figure 10), which shows, for each data source, the forecasted (max) number of possible interactions from the selected entity. Moreover, it is possible to select one of three expansion methods. The first will only add the interactions between the selected node and its interactors (Figure 10a); the second will allow to also add the interactions between the node interactors and any other node present in the network (Figure 10b). The last option will run an additional routine that will keep only those interactors that have at least two interactions with any other node of the network (Figure 10c).

## Conclusion and future work

In this article, we presented the RING database, a complex data aggregation framework that was designed to organize, standardize and integrate omics data from several available public data repositories. The RING can be accessed through a web application that allows researchers to explore potentially millions of regulatory interactions through a user-friendly interface.

The RING project is only the beginning of the longer-term objective of being able to efficiently explore the whole human genome. In future releases we plan to

- add the possibility to overlay the network with other information like expression data, tissue types or phylogenetic conservation data;
- run basic dynamic simulations of the network behavior, for example to investigate the expression profiles of all the nodes of the network starting from the expression of a subset of nodes;
- add batch execution capabilities to allow for more data-intensive queries;
- add the possibility to calculate basic graph metrics like nodes degree and betweenness, centrality, as well as shortest paths between pairs of nodes to better investigate possible indirect regulations between nodes; this last step requires to work with a graph representation of the data and not from directly with the SQL database.

*Conflict of interest*. None declared.

## Supplementary Material

add_mat_baz108

## Notes

**Database URL**: https://precious.polito.it/theringdb/
