# Supplementary material for: ‘One DB to rule them all’—the RING: a Regulatory INteraction Graph combining TFs, genes/proteins, SNPs, diseases and drugs
Source: Database (Oxford). 2019 Nov 4;2019:baz108. doi: 10.1093/database/baz108 (PMC6827393; doi:10.1093/database/baz108)
Supplement: add_mat_baz108 [file add_mat_baz108.zip › add_mat_baz108.docx]

# Introduction

Premise. In this document we refer to “entity” to generally describe a network node, which may be a gene/protein, tf, miRNA, snp, drug or disease . All the reported examples have not any biological meaning, they are reported only to provide use cases of the RING database.

# Example 1 – Single Entity Regulome

Search for all interactors of a single entity.

In this example we first introduce a gene of interest: ALCAM.


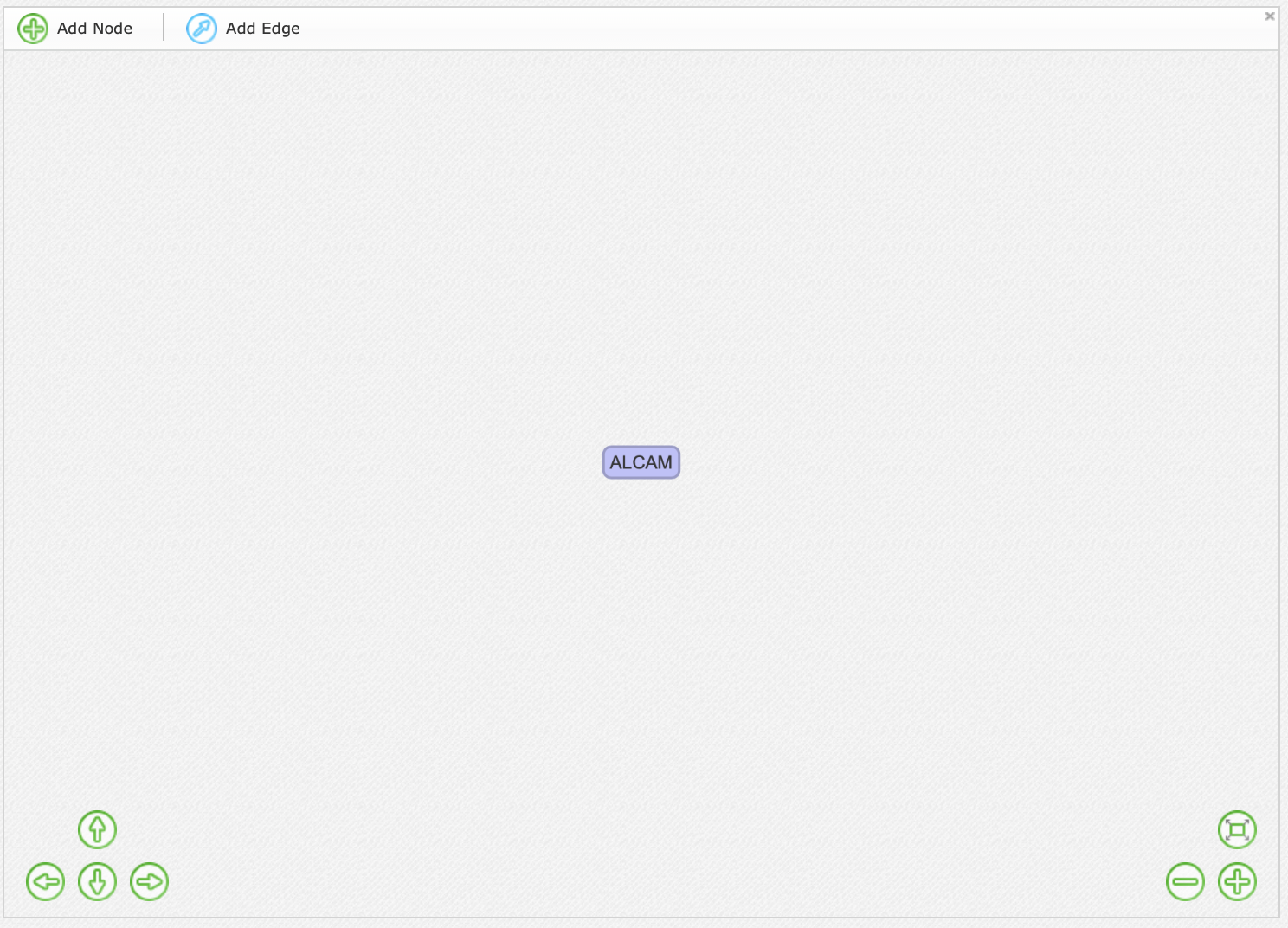


Then we select on top filters all the entity types to connect, i.e., GENE, TF, MIRNA, SNP, DRUG, DISEASE.


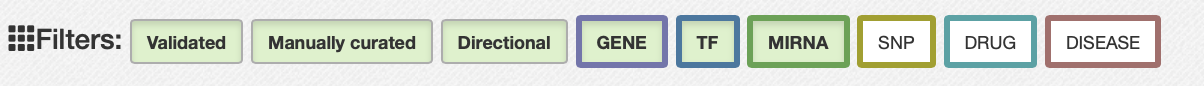


Then we manually select ALCAM in the network by clicking on it and perform an Expand entity 1-level procedure choosing the default option in the Utils selectbox and clicking on GO button.

A modal appears to further refine our selection of data sources.

The default configuration allows to only retrieve the most reliable data on regulation, according to the scope of the designed analysis users may take advantage of very reliable information, by filtering in only validated, manually curated and directional information. In order to possibly enlarge the result space and provide larger although less reliable results, some predefined filter may be turned off, which in turn result into the activation of new databases.

In this example, since SIGNOR does not provide any Protein interaction (PPI), a user may consider to disable the Directional filter in order to allow some PPI, which are mostly undirected.


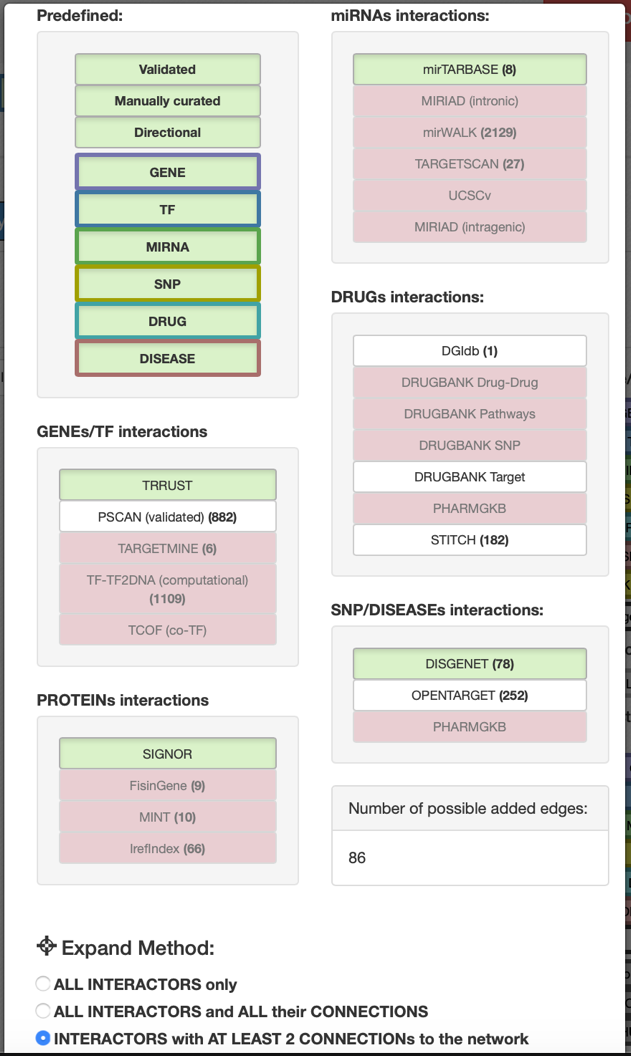

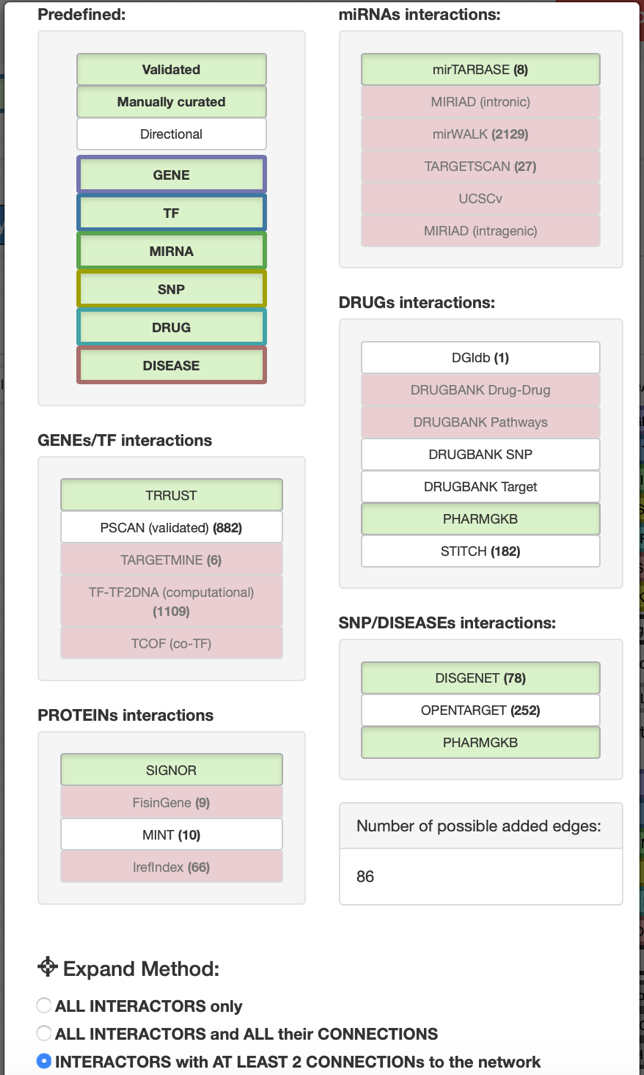


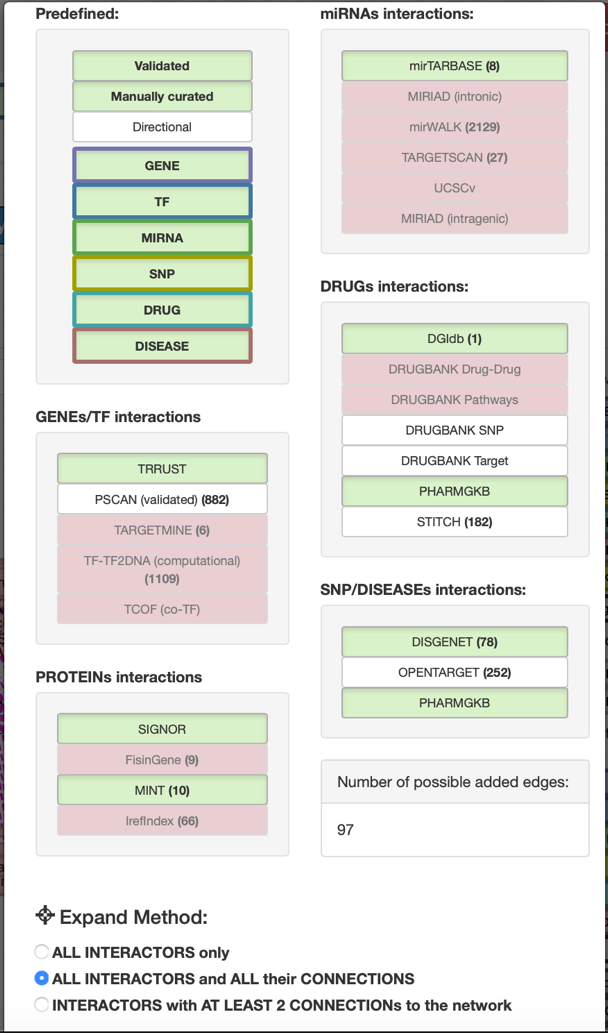
After that, some previously filtered out DB (i.e. red background) become available (i.e. white background). Available databases can be selected (i.e., green background) to be included in the query.

We then choose MINT and DGIdb, in order to have the most heterogenous regulome of the ALCAM gene.

When confident with the DB choice, the last thing to decide is the Expand Method.

Since in this example we are interested in the complete regulome we go with the second choice. So that the system will return the whole ALCAM regulome along with all possible interconnections among ALCAM’s regulator.

We can finally click on Expand Node and let the system work.

In about 20/30 seconds we obtain the resulting network. We first apply a “original” layout in order to separate nodes and provide a better insight.


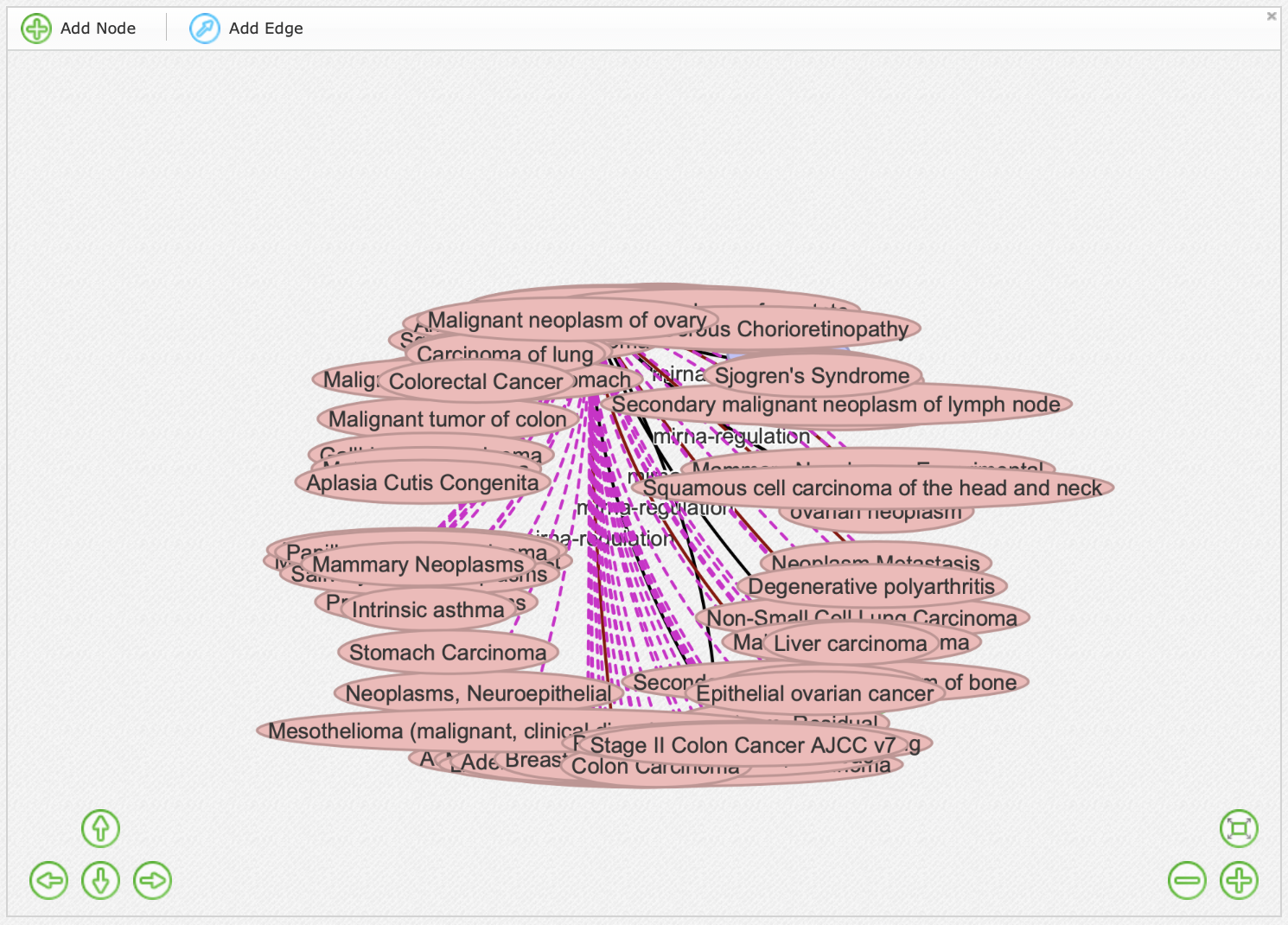

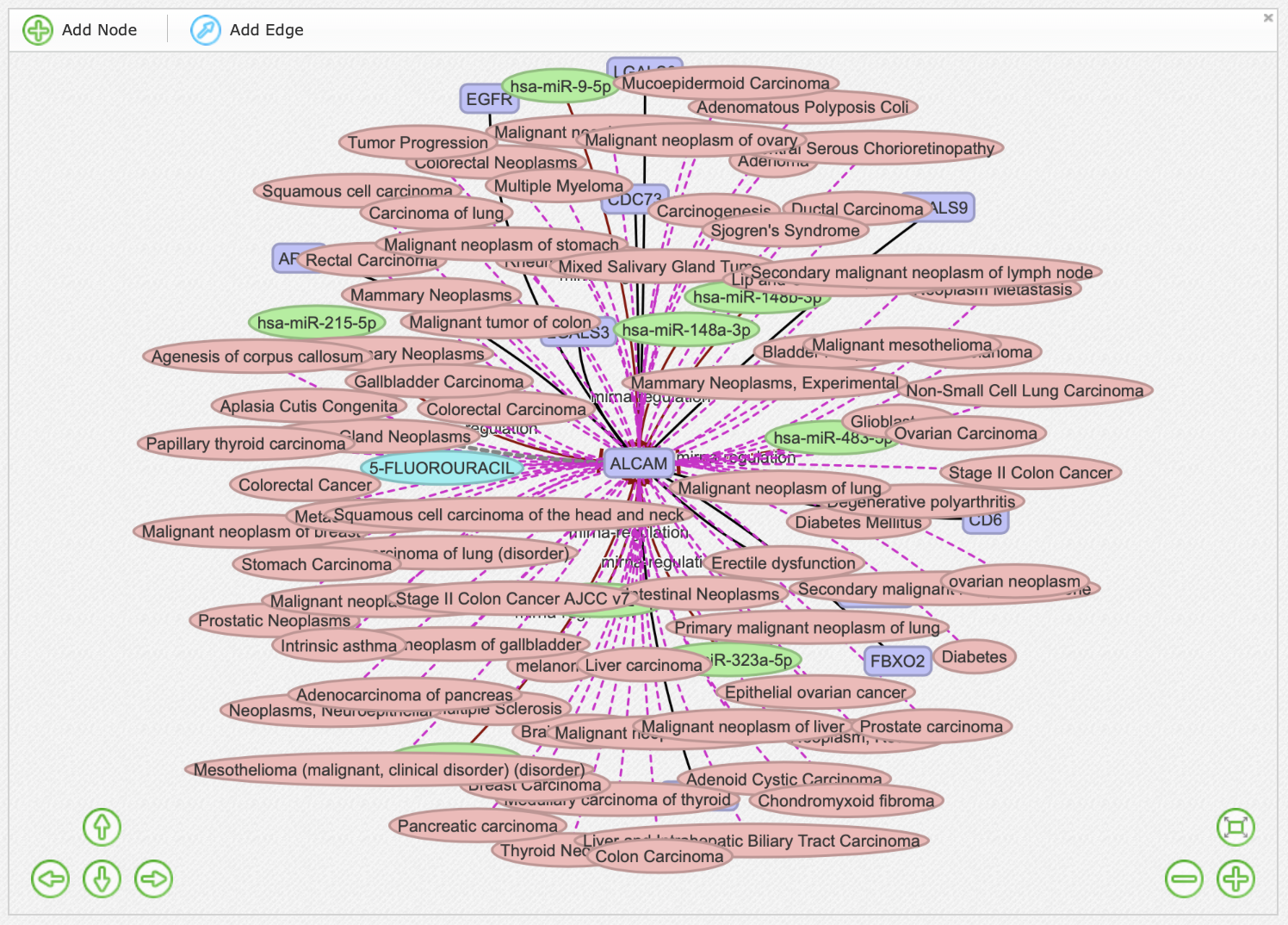


The resulting network thus shows the regulome of the ALCAM gene according to the list of chosen allowed entity and interaction DBs. The regulome includes miRNAs, TFs and Drugs.

# Example 2 – Infer a network starting from some entities

Connect sparse entities in order to provide a meaningful regulatory linkage among them.

Let’s say, for example, we are interested in some detailed information regarding LRP6 mediated regulation in mTOR pathway, see <https://www.genome.jp/kegg-bin/show_pathway?hsa04150>


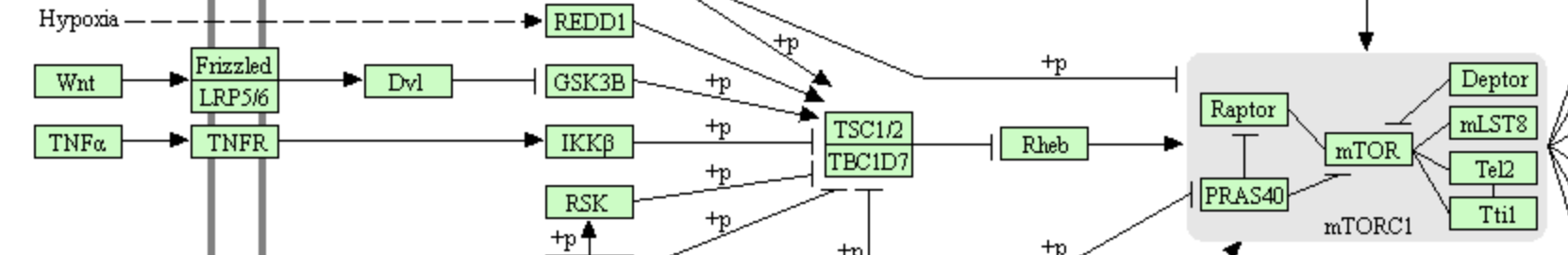


and to understand their role in regulating main entities such as Raptor and mTOR.


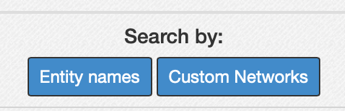
In order to do that we have to click on the Entity names button and provide, as input, a comma separated list of interesting entities as:

LRP6, RAPTOR, MTOR


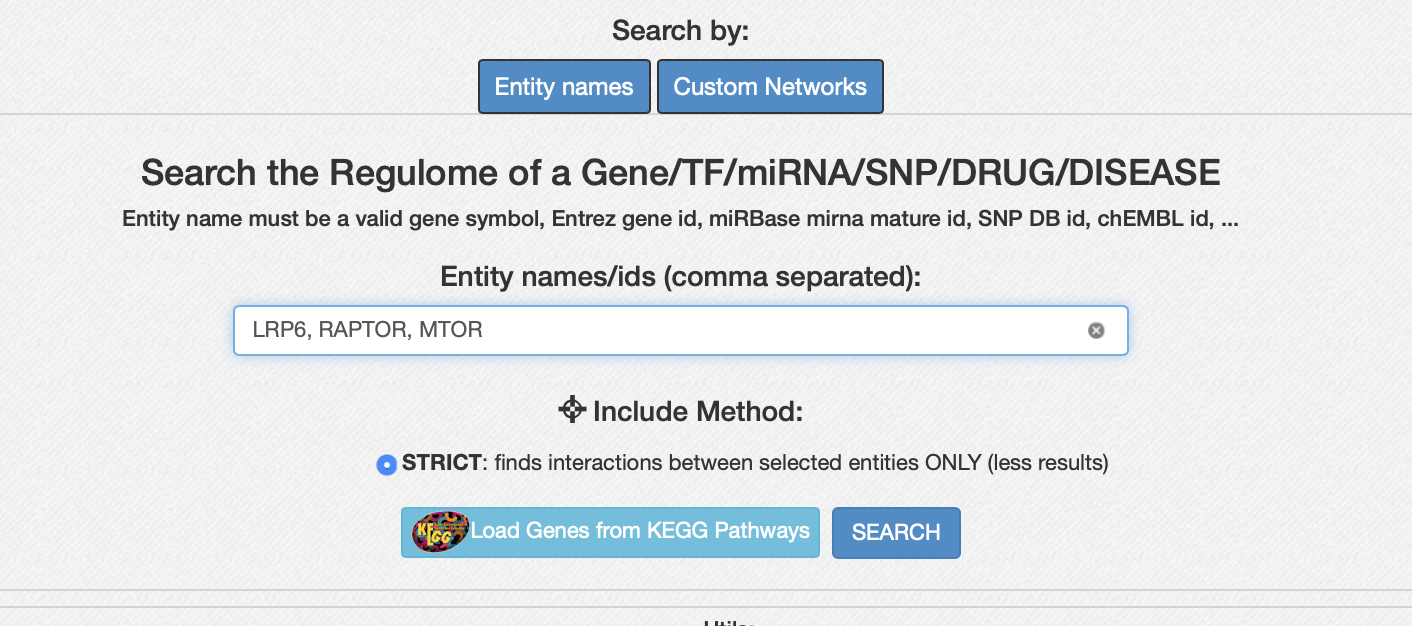


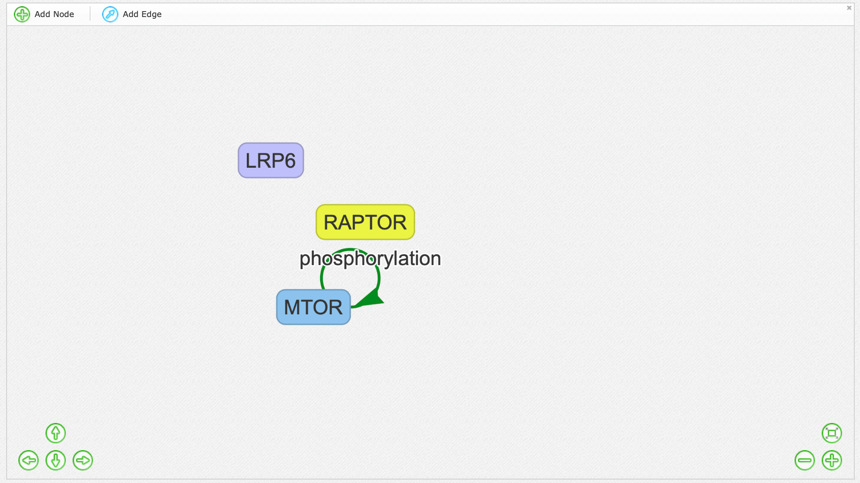
By clicking search we introduce the three new nodes into the network and after confirming they have been correctly recognized (i.e., they are colored according to valid entities in the network; pale blue for TF, pale purple for genes, and so on..) we can start to analyze them. In this example we notice that raptor is colored in yellow, which means it has not been correctly recognized due to a name misspell. In order to correct it we need to change its name to RPTOR which is one of the valid aliases of the gene itself.


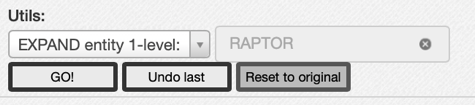


We can reset the network to original

Perform the correction and start again.

LRP6, RPTOR, MTOR


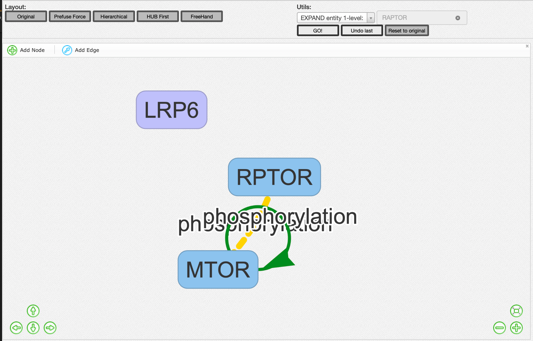


Now the three genes look correctly recognized, and we can further proceed in our analysis.

We are interested in providing a linkage among those entities in order to better understand the signaling cascade which may take place between LRP6 and other main actors on the mTOR pathway.


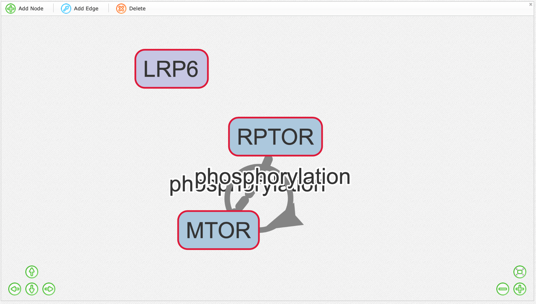


To do that we select all three entities (by pressing ctrl/cmd and clicking on all of them, or by pressing “Select Layer 0” button). A red border around the entities confirm us they are properly selected, moreover they are properly listed in the Utils box.


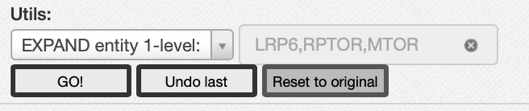


We can GO with the Expand entity 1-level.

We let all filter in default mode in order to only extract the most reliable information and we deselect miRNA in order to limit the signal cascade to only gene and protein.

We finally choose “**ALL INTERACTORS and ALL their CONNECTIONS**” to identify possible regulators that also have multiple targets on the network. This allows to possibly maximize the regulatory meaning of newly identified nodes. We can click EXPAND.


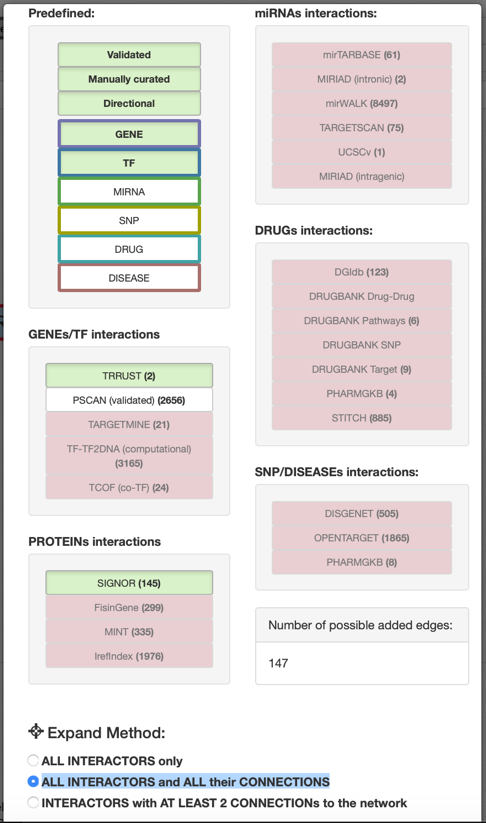


This expansion may take up to a couple of minutes to finish. Just wait until the progress bar indicate that the procedure has finished.


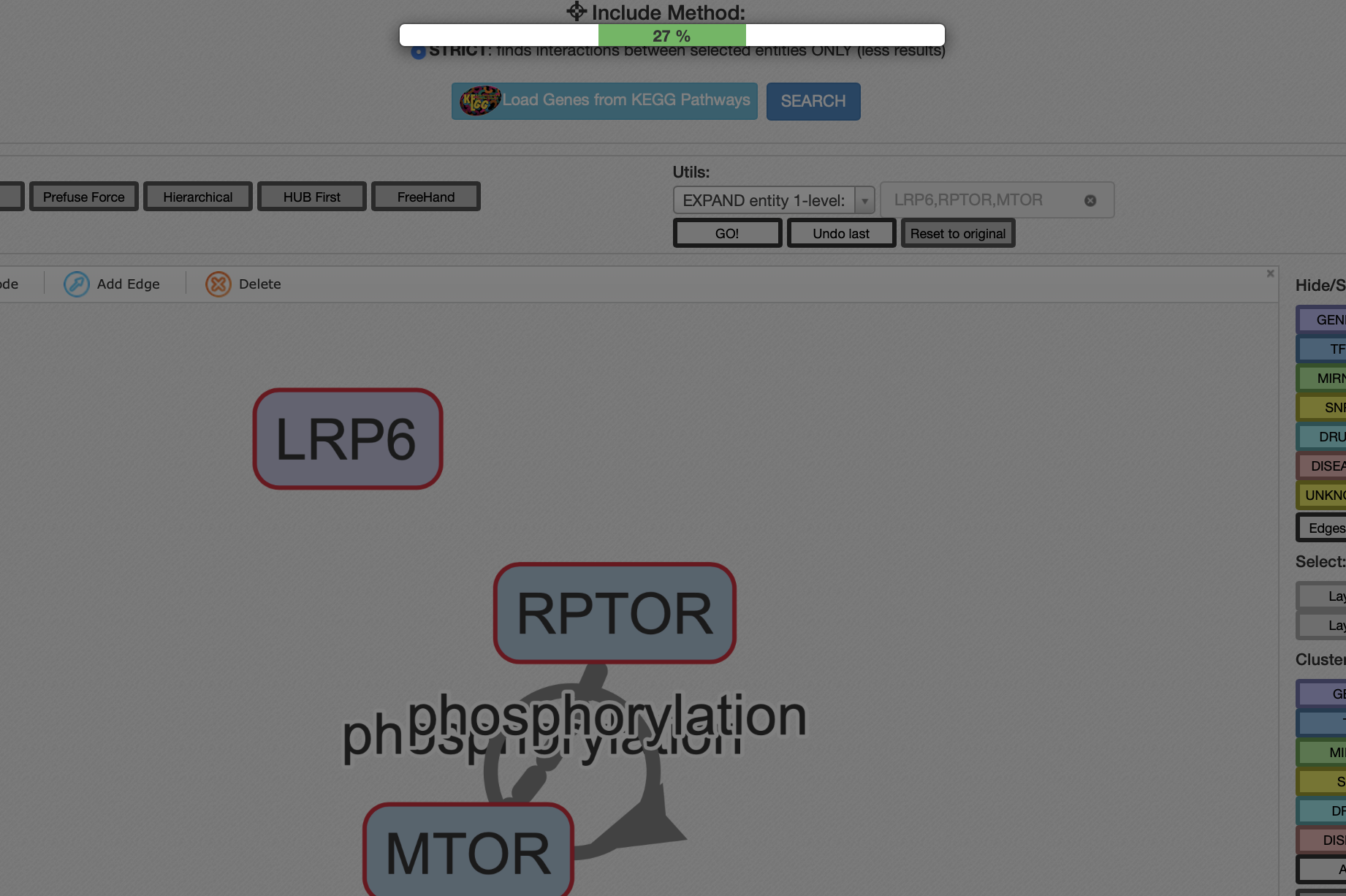


After the procedure terminates, we can apply a Prefuse Force layout in order to separate nodes and present them in a comprehensible fashion.


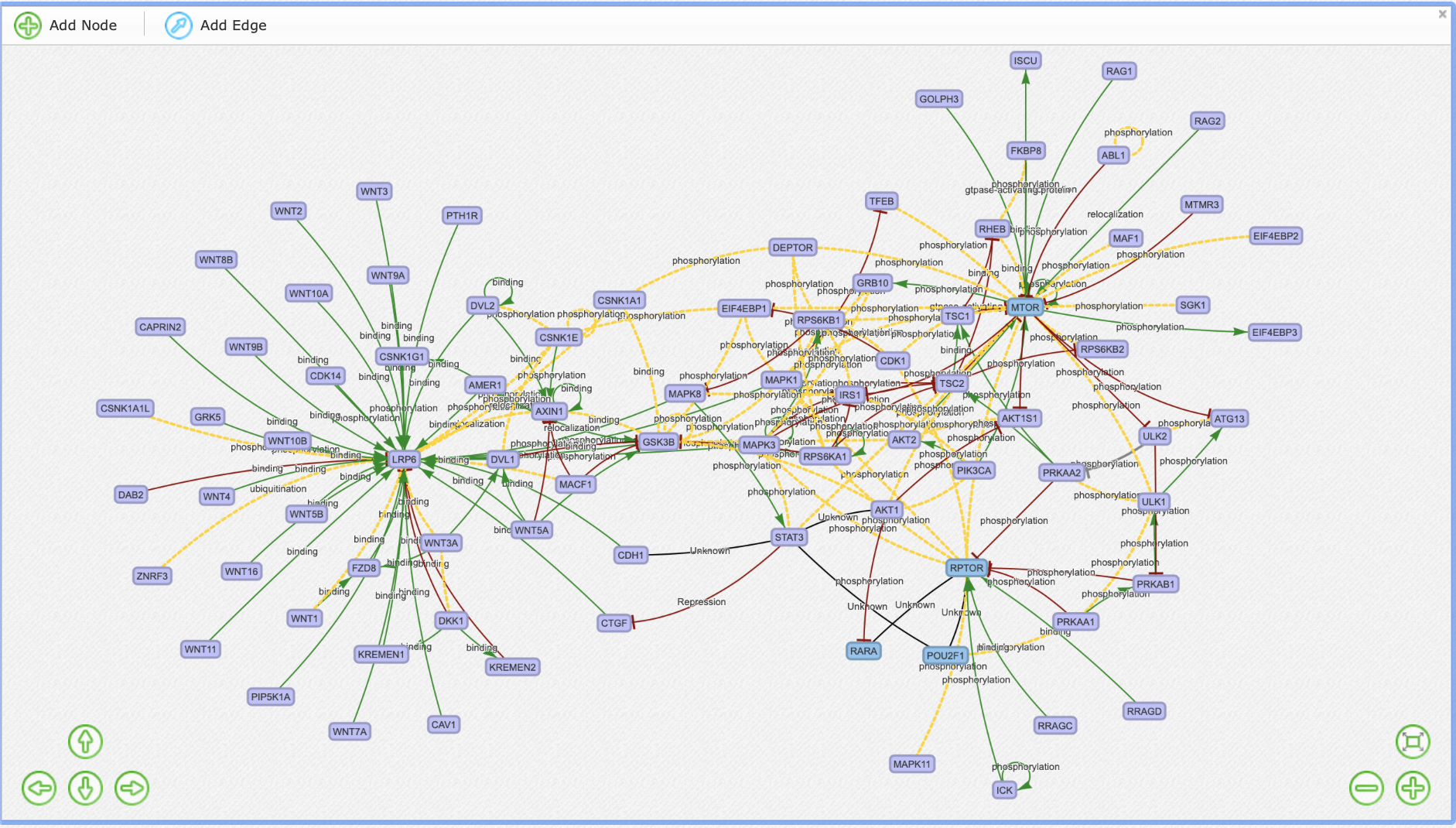


Interestingly from only three nodes the system extracts a large and well-connected network, which also depicts the large WNT cluster preceding LRP6 and the GSK3B bridge, which looks very important for the signal transmission, along with DVL, TSC and RHEB


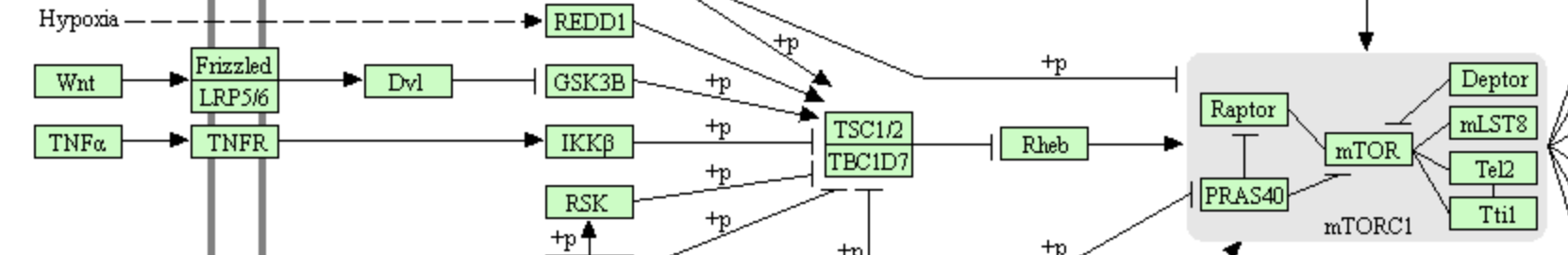


Researcher could, at this point, further enlarge the neighborhood of GSK3Bto possibly identifying further possible regulation. Let’s for instance search for some drugs


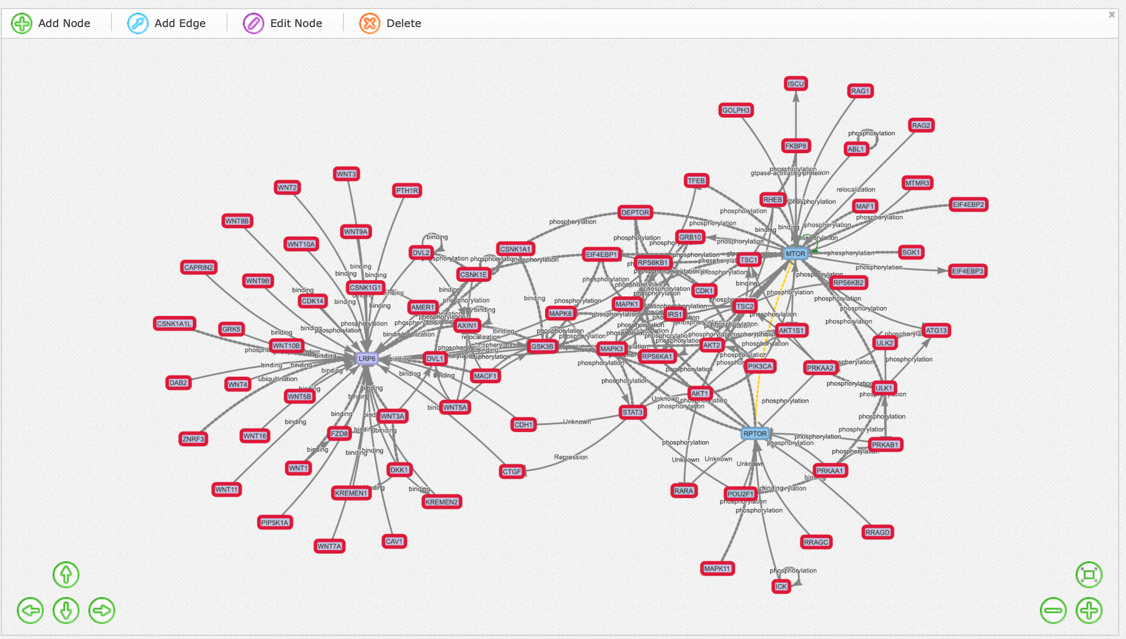


First, select all the newly introduced nodes (Select: Layer 1)


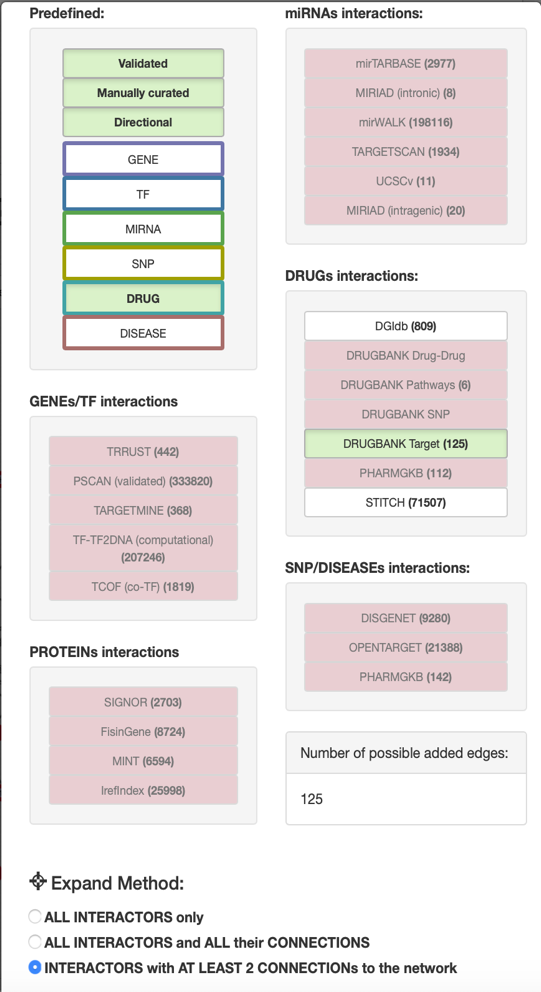


Then configure the filters in order to only search for DRUGs. And further limit DB sources to Drugbank in order to keep low the number of results.

The chosen expand method will be **“INTERACTORS with AT LEAST 2 CONNECTIONs to the network**“ in order to only show drugs that have multiple targets on the network. Which would possibly reflect a larger or more robust effect on this signaling cascade.

This procedure is pretty fast and after few seconds we get back our network with drugs included


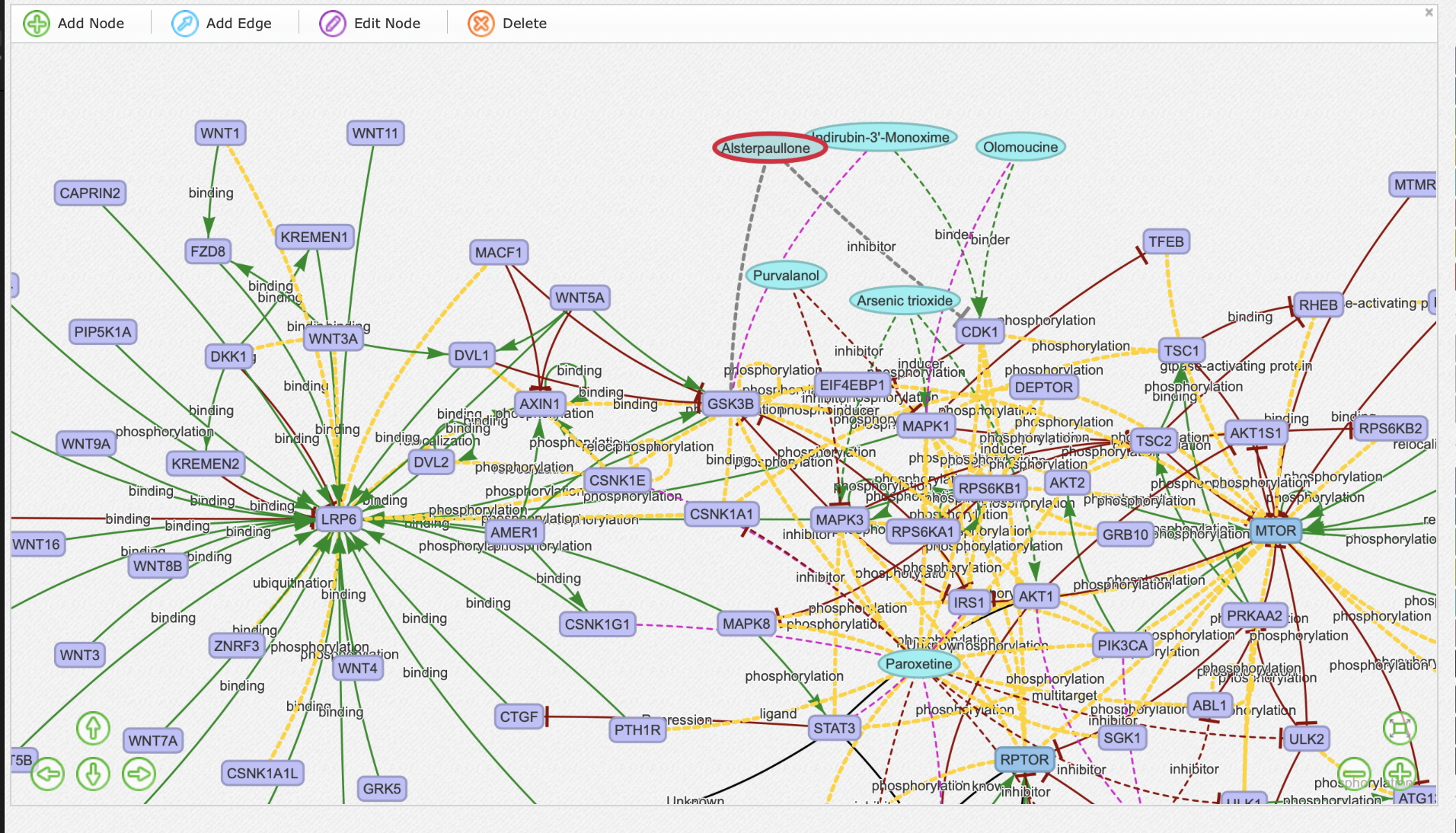


Few drugs now appear to have direct interaction on the bridge we noticed in the previous step in particular **Alsterpaullone** and **Indirubin-3'-Monoxime,** which would be the first targets of further network expansion to better highlight their possibly competing targets.

.
